# 94. Rapid Diagnostic Testing is Associated with Decreased Broad Spectrum Antibiotic Use in Streptococcal Blood Stream Infections

**DOI:** 10.1093/ofid/ofad500.010

**Published:** 2023-11-27

**Authors:** Tyler Tate, Alex Viehman, Ryan K Shields

**Affiliations:** University of Pittsburgh Medical Center, Pittsburgh, PA; University of Pittsburgh, Pittsburgh, Pennsylvania; University of Pittsburgh, Pittsburgh, Pennsylvania

## Abstract

**Background:**

Rapid diagnostic testing (RDT) allows antimicrobial stewardship programs (ASP) to intervene early in the setting of sepsis and septic shock leading to lower rates of death. Combining RDT with ASP results in faster antibiotic de-escalation compared to RDT alone; however, the utility of this approach for Streptococcal bacteremia is unproven. The objective of this study was to evaluate the impact of RDT with real-time ASP intervention on blood cultures growing *Streptococcal* species.

**Methods:**

Retrospective, observational study comparing patients with *Streptococcus spp*. bacteremia over two 6-month periods. During the pre-RDT period, patients were prospectively followed by ASP who provided recommendations based on gram-stain and organism identification results. During the post-RDT period, blood cultures were tested by GenMark ePlex BCID-GP that identifies *S. agalactiae*, *S. anginosis* group, *S. pneumoniae*, *S. pyogenes* and *Streptococcus spp*. within 90 minutes which were reported directly to ASP for recommendations on antibiotic discontinuation, de-escalation, and additional diagnostic testing.

**Results:**

94 patients were included in the analysis, 50 pre- and 44 post-RDT. Across cohorts, patient demographics, clinical characteristics, and severity of illness were comparable (**Table 1**). The median ages were 58 and 56, 62% and 68% were men, and a total of 12% and 16% were immunocompromised. The mean qSOFA scores were 1.9 and 2. The most common *Streptococcus spp*. was *S. mitis* (**figure 1**). Prior to RDT, the median time to antibiotic de-escalation was 88 hours. Following implementation of RDT, median time to de-escalation was 42 hours (P=0.002). De-escalation to targeted therapy improved from 50% to 86% in post-RDT (P< 0.01). Transition to oral antibiotics increased from 14% to 30% (P=0.08). There were no differences in hospital re-admission or acute kidney injury. A trend toward decreased 30-day mortality was identified in the post-RDT period (P=0.06).

**Figure:**
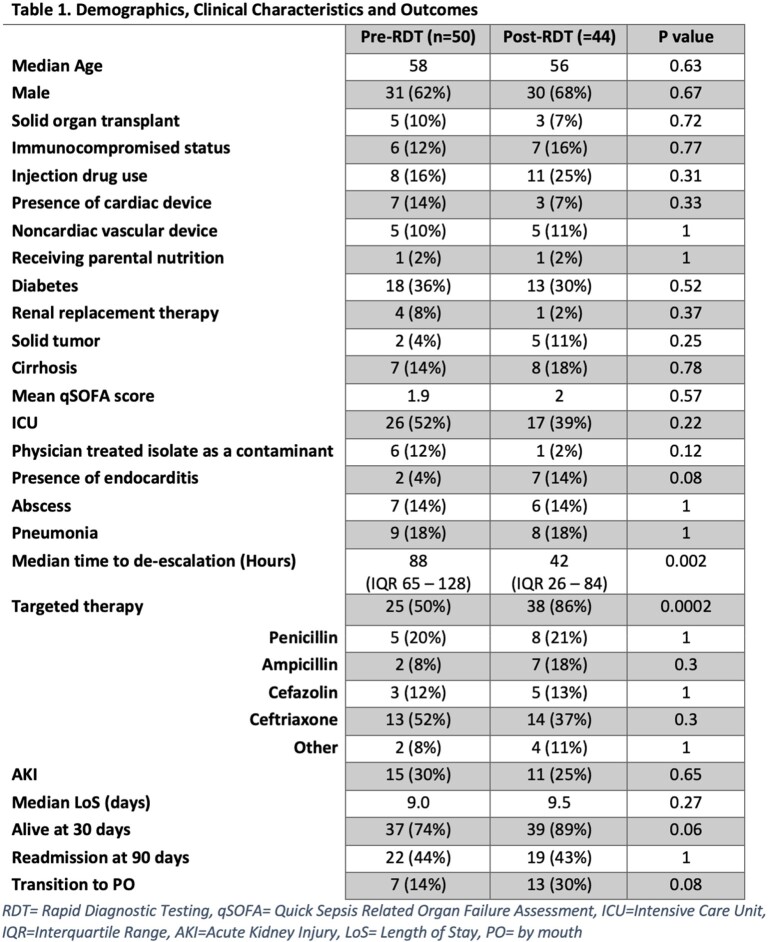

**Figure:**
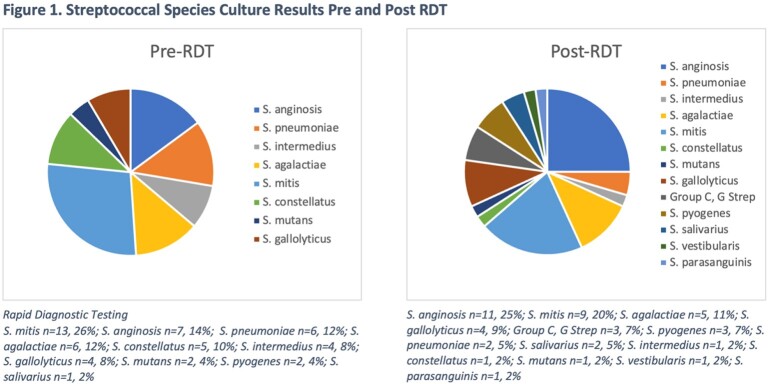

**Conclusion:**

Our study highlights the impact of RDT with ASP intervention on clinical outcomes for patients with *Streptococcal* bacteremia. We noted significant reductions in broad-spectrum antibiotic use and a trend towards lower mortality. Larger studies are needed to demonstrate the full potential of this approach.

**Disclosures:**

**Ryan K. Shields, PharmD, MS**, Allergan: Advisor/Consultant|Cidara: Advisor/Consultant|Entasis: Advisor/Consultant|GSK: Advisor/Consultant|Melinta: Advisor/Consultant|Melinta: Grant/Research Support|Menarini: Advisor/Consultant|Merck: Advisor/Consultant|Merck: Grant/Research Support|Pfizer: Advisor/Consultant|Roche: Grant/Research Support|Shionogi: Advisor/Consultant|Shionogi: Grant/Research Support|Utility: Advisor/Consultant|Venatorx: Advisor/Consultant|Venatorx: Grant/Research Support

